# Institutional Dentistry; Methods; Results

**Published:** 1913-01-15

**Authors:** Frederick A. Keyes

**Affiliations:** Boston, Mass.


### INSTITUTIONAL DENTISTRY; METHODS; RESULTS.

BY FREDERICK A. KEYES, D.M.D., BOSTON, MASS.

At the present day there is great agitation by medical and dental men for the improvement of the oral conditions existing among the public school children. The dental profession has always been cognizant of the importance of oral cleanliness in relation to the health of the human body, but a few medical men with whom I have talked seemed rather skeptical of its importance. I have therefore gathered together carefully these statistics, showing the relation of the condition of the mouth to infectious diseases.

I hope that this evidence of the elimination of infectious disease by having the mouth and teeth treated will arouse the medical profession to the great importance of oral sepsis. Even though the results obtained by two years' dental treatment may seem merely a coincidence, I am confident that such is not the case, and that results equally good are being obtained by all dentists treating adolescent children.

In November, 1910, I was requested by Mother Rose, Superior of St. Vincent's Orphan Asylum, Camden street, Boston, to visit the institution to see what steps would be necessary to establish a dental infirmary for the care of the children's teeth. Within a week a fully equipped infirmary was established on the third floor of the home. A large bathroom, having all the desired requisites, such as running, hot and cold water, hardwood floor, one large window, plenty of light and sun, was the site chosen for the operating room. Owing to the pecuniary limitations, a second-hand chair and engine were purchased, together with an essential supply of dental paraphernalia (brackets, brushes, etc.), all of which netted $125.

In order to demonstrate the cost of establishing dentistry in an institution for a limited sum, $125, the items are given below:

| | |
|---|---|
| One chair and engine, sterilizer, brushes, etc., for one year, cements and amalgam for one year, medicinals, miscellaneous, cotton rolls, napkins, card system, sandpaper, etc., initial sum | $125.00 |
| Supplies for 18 months | 66.63 |
| | $191.63 |

Instruments were supplied by the dentists doing the work.

Every three months the children were lined up, and passed by the dentist one after the other, and a more thorough examination than that of the first day was made, using a separate mouth stick on each child. Each child was given a red, blue or green card, according to the condition found in her mouth. These cards were distributed by the dentist's assistants at his call. The red cards signified an acute abscess; blue, exposed pulps; green, dirty teeth or sloughing gums. It took just two hours to examine the teeth of over two hundred children in this manner.

STATISTICS OF WORK DONE FOR FOURTEEN MONTHS.

| | |
|---|---|
| Total number of different patients examined | 349 |
| Total number of cleanings | 272 |
| Total number of cement fillings | 35 |
| Total number of amalgam fillings | 72 |
| Total number of cement-amalgam fillings | 69 |
| Total number of temporary fillings | 130 |
| Total number of oxyphosphate copper fillings | 9 |
| Total number of teeth extracted (temporary) | 290 |
| Total number of teeth extracted (permanent) | 131 |
| Total number of abscesses opened | 42 |
| Total number of gums cut for permanent teeth | 19 |
| Total number of plastic operations | 3 |
| Total number examined and treated | 1,421 |
| Total number of fillings (including temporary) | 315 |
| Total number of teeth extracted (including temporary) | 421 |
| Total time spent, hours | 210 |

The rapidity of the decay of deciduous teeth over permanent teeth was marked. Whether or not the decay of deciduous teeth is purely a physiological action is not known, but to observe the ravages in the mouths of many of these small children, it would be hard to believe that it was solely a pathological condition. One reason for this rapid decay is the fact that in the physiological process of the absorption of the roots, the blood and nerve supply becomes diminished and the teeth become more susceptible to the multitude of bacteria always present in the mouth. Out of three hundred children, there was not one per cent who had even had any dental care, and there was not one child but needed treatment.

In the extraction of permanent teeth, a tooth was not sacrificed unless the crown was all decayed to the roots.

Cocaine one-half per cent was the only anesthetic used, and that only in extraction of permanent teeth. Another example of economization of time was in the method of use of cocaine, six or seven children having the cocaine injected one after another, so that when the gums of the seventh child were cocainized, the first child's gums were anesthetized for extraction.

When the work was completed in the mouth of a child, the tooth treated was called to the attention of one of the assistants, who marked it down on the card system. After a month, without my pointing to picture of a tooth on the card, the assistant could mark it alone.

Twenty-five per cent of the children required orthodontia. Of this amount all had either adenoids or tonsils, and were recommended to Dr. Drummey, the house physician, for operation.

The following interesting statistics show the relation of oral prophylaxis to infectious diseases:

RECORD IN REGARD TO INFECTIOUS DISEASES.

| | 1907 1908 | 1908 1909 | 1909 Nov., 1910 | Nov., 1910 April, 1911 | April, 1911 May, 1912 |
|---|---|---|---|---|---|
| Diphtheria | 6 | 2 | 1 | 0 | 0 |
| Mumps | 8 | 3 | 10 | 4 | 0 |
| Scarlet fever | 17 | 8 | 12 | 8 | 0 |
| Pneumonia | 3 | 5 | 4 | 6 | 0 |
| Measles | 24 | 50 | 40 | 25 | 0 |
| Tonsilitis | 19 | 16 | 8 | 3 | 0 |
| Whooping cough | 7 | 2 | 2 | 0 | 0 |
| Chicken pox | 15 | 17 | 10 | 6 | 0 |
| Typhoid | 0 | 0 | 0 | 0 | 0 |
| Croup | 4 | 0 | 0 | 0 | 0 |
| Spinal meningitis | 0 | 0 | 0 | 0 | 0 |
| Scarletina | -- | -- | 0 | 0 | 0 |
| Bright's (acute) | -- | -- | -- | -- | 0 |
| Hemorrhage | -- | -- | -- | -- | 0 |
| Tuberculosis of eye | -- | -- | -- | -- | 1 |
| Tuberculosis of lungs | -- | -- | -- | -- | 1 |
| | 103 | 103 | 87 | 52 | 2 |

In the years 1905-1906, the home was in quarantine for over three months,—an epidemic of scarlet fever of over 75 cases.

A comparison of these statistics will show that in six months after work was begun at the institution the ratio of infectious diseases was reduced 59 per cent; and that in the subsequent year this ratio was reduced to approximately 2 per cent, a most notable decrease.

Is this absolute elimination of disease for a period of twelve months a coincidence? It may be so. Other factors may have brought about this remarkable condition. But certainly no such condition ever existed in St. Vincent's Asylum prior to the installation of a dental infirmary. Of course, two years more will produce much more conclusive evidence, although one may see even now that the benefit of dental work is not over estimated, as far as the elimination of infectious diseases is concerned.—*Boston Medical Journal of Oral Hygiene.*

